# An Experimental Rat Model for Simultaneous Induction of Peripheral Neuropathy and Myelotoxicity by Docetaxel Administration: Evaluating the Protective Role of Dimethyl Fumarate

**DOI:** 10.3390/ijms26125859

**Published:** 2025-06-19

**Authors:** Sebastian Cubides-Cely, Alexander David Castro, Pablo Prado-Guevara, Julio César Mantilla-Hernández, Mario Negrette-Guzmán

**Affiliations:** 1 Pharmacology and Metabolism Research Laboratory, Universidad Industrial de Santander, Bucaramanga 68002, Colombia; 2 Department of Pathology, Universidad Industrial de Santander, Bucaramanga 68002, Colombia

**Keywords:** docetaxel, peripheral neuropathy, nociception, myelotoxicity, neutropenia, dimethyl fumarate

## Abstract

Docetaxel is extensively used for treating different types of cancer; however, its clinical efficacy is primarily limited by myelotoxicity and peripheral neuropathy, adverse effects that often lead to treatment discontinuation. This study aimed to establish a preclinical model in Wistar rats for the simultaneous induction of myelotoxicity and peripheral neuropathy associated with docetaxel administration, enabling the evaluation of potential chemopreventive agents. Four distinct docetaxel administration schemes were assessed by performing behavioral nociceptive tests and complete blood cell counts. After establishing the damage model (5 mg/kg/week docetaxel for six weeks), we co-administered 100 mg/kg/week oral dimethyl fumarate to assess its protective effect. Dimethyl fumarate attenuated docetaxel-induced hyperalgesia, likely through preserving normal nerve fiber density in sciatic nerves, but neutropenia was not significantly mitigated. An alternative regimen with additional pre-administered doses of dimethyl fumarate showed a trend toward neutropenia attenuation and suggested an interesting inhibition of docetaxel-induced rat vibrissae loss. Chou-Talalay isobolographic analyses on prostate cancer cell lines revealed that dimethyl fumarate does not impair the therapeutic effect of docetaxel at most combination ratios evaluated; rather, synergistic effects were observed. This experimental model proved useful and will facilitate further research into the protective role of dimethyl fumarate and other potential chemoprotective agents.

## 1. Introduction

Docetaxel is a semi-synthetic taxane approved by the U.S. Food and Drug Administration (FDA) for the treatment of several malignancies, including breast, ovarian, lung, and prostate cancer [1,2]. The anticancer activity of this paclitaxel analog is mediated by its binding affinity for beta-tubulin subunits of microtubules, thereby inhibiting microtubule depolymerization and stabilizing their structure. This stabilization induces cell cycle arrest at the mitotic phase, subsequently leading to cell death [3]. Additionally, docetaxel promotes intrinsic apoptosis by suppressing the expression of anti-apoptotic proteins such as BCL-2 and BCL-X and inhibits cancer proliferation through downregulation of androgen receptor signaling [4]. Clinical efficacy of docetaxel depends on drug concentration and exposure time; however, it is limited by associated adverse effects, primarily peripheral neuropathy, febrile neutropenia, alopecia, infusion reactions, fatigue, fluid retention, pneumonitis, and gastrointestinal complications [5,6].

Peripheral neuropathy induced by taxanes manifests clinically as a myalgia and arthralgia syndrome, predominantly in the legs, hips, and lower back [7]. Precise pathophysiological mechanisms of taxane-induced neurotoxicity remain elusive; however, evidence suggests that taxanes induce stabilization of axonal microtubules, altering their polarity and disrupting axonal transport of mitochondria and other essential cellular components [7]. These disruptions ultimately compromise axonal integrity, leading to nerve degeneration and altered neuronal function [7]. Recent clinical data underline the substantial prevalence and variability of peripheral neuropathy associated with different taxanes and administration schemes [8]. In the case of docetaxel, studies have documented incidences of up to 64%, with severe cases accounting for approximately 7.4% [7]. Neither the American Society of Clinical Oncology (ASCO) nor the European Society for Medical Oncology (ESMO) endorses specific preventive or therapeutic agents for chemotherapy-induced peripheral neuropathy. Clinical trials report moderate symptomatic relief with duloxetine, especially effective in neuropathies associated with platinum-based chemotherapeutics rather than taxanes [9]. Other pharmacological strategies, such as tricyclic antidepressants and anticonvulsants, have yielded inconsistent outcomes [10,11]. The need for effective chemoprotective strategies is urgent.

Severe neutropenia is another significant complication of chemotherapy that frequently forces dose reductions, potentially compromising anticancer efficacy [12]. Guidelines addressing the prevention of chemotherapy-induced neutropenic infections are well-established [13], advocating the prophylactic use of antibiotics as standard treatment for febrile neutropenia in low-risk patients [14]. However, some alternatives against chemotherapy-induced neutropenia have recently been provided. For example, granulocyte colony-stimulating factors (G-CSFs), such as pegfilgrastim, have shown promise in patients by enhancing neutrophil production [15]. Additionally, the immunomodulatory agent plinabulin, with hematopoietic protective effects independent of G-CSF mechanisms, has shown efficacy against docetaxel-induced neutropenia in animal models [12,16]. Cellular and molecular mechanisms of taxane-induced myelotoxicity that lead to neutropenia have not been fully elucidated.

Dimethyl fumarate, the methyl ester of fumaric acid (a compound derived from the plant *Fumaria officinalis*), is an FDA- and European Medicines Agency (EMA)-approved drug for the treatment of relapsing–remitting multiple sclerosis and psoriasis. It is considered a type of indirect antioxidant, as it promotes the activation and stabilization of nuclear factor erythroid 2-related factor 2 (Nrf2) [17,18]. Recent findings further confirm the capacity of dimethyl fumarate to mitigate neuroinflammation by activating Nrf2 and concurrently inhibiting the nuclear factor kappa B (NF-κB) inflammatory pathway in microglial cells, suggesting potential therapeutic efficacy for other neurodegenerative conditions [19]. Dimethyl fumarate enters the body orally and dissociates in the intestine, where it subsequently interacts with immune cells in the blood circulation and crosses the blood–brain barrier into the central nervous system [20]. Previously, the attenuation of neuropathic pain induced by the taxane paclitaxel in Wistar rats was reported using oral dimethyl fumarate, seemingly mediated by an increase in the Nrf2 signaling pathway and a reduction in pro-inflammatory cytokines [21]. In addition, dimethyl fumarate has been studied as an antitumor chemotherapeutic in experimental models [22,23] and clinical trials with promising outcomes [24]. All the above motivates an interest in assessing dimethyl fumarate as a potential protective agent not only against docetaxel-induced neuropathy but also neutropenia, with the added value of improving the pharmacological effect against cancer.

Given the limitations of current clinical management strategies, further preclinical research is needed to develop interventions that effectively mitigate docetaxel-induced neuropathy and neutropenia without diminishing therapeutic efficacy. This study aimed to establish a simultaneous docetaxel-induced myelotoxicity and peripheral neuropathy rat model to test potential protective agents, optimizing animal use, and enhancing ethical research standards. Using this experimental model, we first assess the protective role of dimethyl fumarate against docetaxel-induced neutropenia and neuropathic hyperalgesia.

## 2. Results

### 2.1. Simultaneous Induction of Nociceptive Alterations and Neutropenia Using Docetaxel

**Figure 1:**
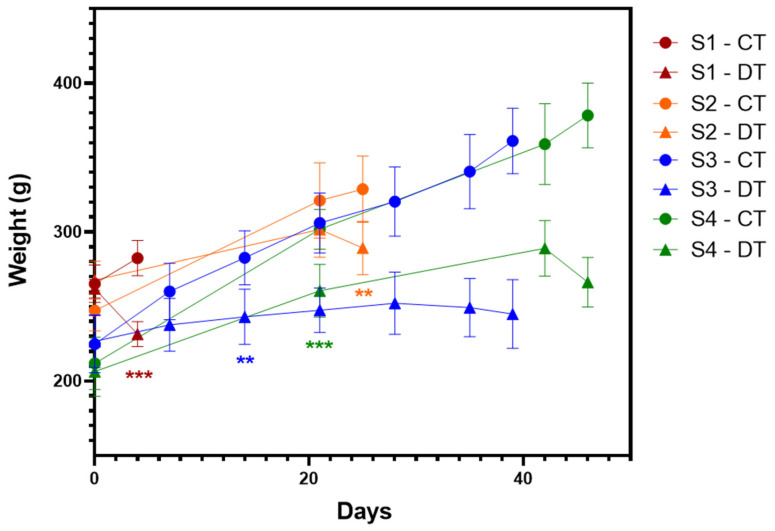
Body weight gain in rats of four administration schemes of docetaxel for simultaneous induction of peripheral neuropathy and neutropenia. Body weight was measured on the days of docetaxel administration for schemes 1 to 4 (S1–4) and the day of euthanasia. CT: control group; DT: docetaxel group. Data are expressed as mean ± standard deviation (SD) and analyzed by *t*-test, n = 8. ** *p* < 0.02, *** *p* < 0.001 vs. CT.

Docetaxel toxicity was evident throughout the treatment as animal behavior and feeding habits changed. Docetaxel-treated rats (DT group) showed a significant lag in body weight gain compared to the control rats (CT group), mainly in the docetaxel administration schemes 3 and 4 (S3 and S4 in Figure 1).

**Figure 2:**
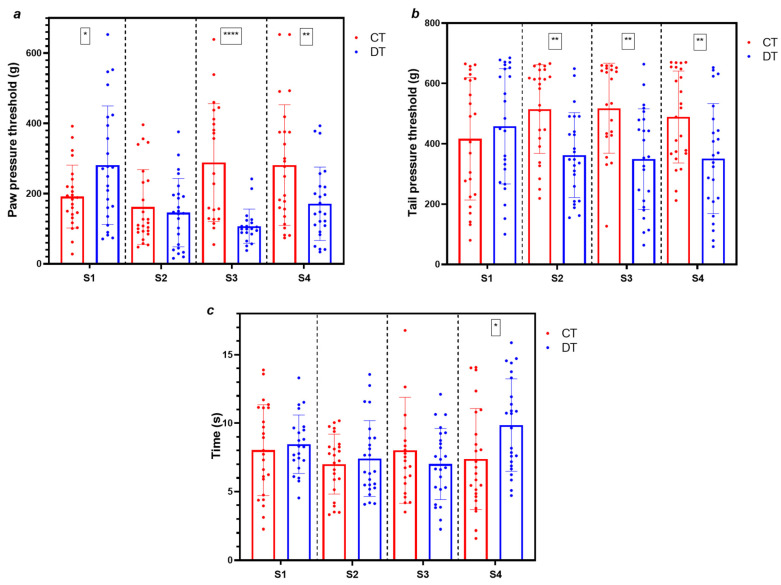
Mechanical and thermal nociceptive thresholds in rats of four administration schemes of docetaxel for simultaneous induction of peripheral neuropathy and neutropenia. All tests were conducted four days after the last docetaxel dose. Values for pressure thresholds in the paw (**a**) and tail (**b**) are given in grams (g). Times of latency at 50 ± 0.1 °C for the hot plate test (**c**) are in seconds (s). CT: control group; DT: docetaxel group; S1–S4: schemes 1–4. Data are expressed as mean ± SD and analyzed by *t*-test (paw pressure: S1 and S4; hot plate: S1 and S4), or Mann–Whitney test (paw pressure: S2 and S3; tail pressure; hot plate: S2 and S3) n = 8. * *p* < 0.05, ** *p* < 0.02, **** *p* < 0.0001 vs. CT.

Schemes 1, 3, and 4 showed differences between the groups in the paw pressure test (Figure 2a), including the unusual 47% increase in the DT nociceptive threshold compared to the CT group in scheme 1 (*p* < 0.05). The mean pressure threshold of the DT group in scheme 3 was almost a third of that for the CT group (*p* < 0.0001), while a 39% decrease in paw pressure threshold of DT rats was observed in scheme 4 (*p* < 0.02 vs. CT). No significant differences between groups were observed in scheme 2, despite the total dose being twice the dose in scheme 1.

In the tail pressure test (Figure 2b), the DT group of schemes 2, 3, and 4 displayed significant falls ranging between 28% and 32% in nociceptive thresholds compared to the CT group. Scheme 1 did not show any statistically significant differences between groups for this test. On the other hand, among the four schemes administered, only scheme 4 showed an alteration in the hot plate test (Figure 2c, *p* < 0.05). However, this alteration, akin to the paw pressure test of scheme 1 (Figure 2a), was characterized by increased nociceptive thresholds in the DT group compared to the CT group. None of the remaining treatments with docetaxel (schemes 1–3) gave a significant response compared to the CT group in the hot plate test (Figure 2c).

**Figure 3:**
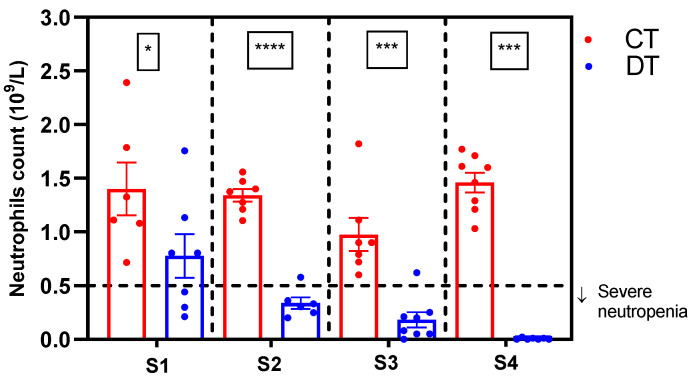
Neutrophil counts in rats of four administration schemes of docetaxel for simultaneous induction of peripheral neuropathy and neutropenia. CT: control group; DT: docetaxel group; S1–S4: schemes 1–4. Data are expressed as mean ± SD and analyzed by *t*-test, n = 8. * *p* < 0.05, *** *p* < 0.001, **** *p* < 0.0001 vs. CT.

Once the pain behavioral tests were completed, euthanasia was performed, and blood was taken for the differential neutrophil count. As shown in Figure 3, all the docetaxel administration schemes led to neutropenia, with neutrophil count decreases of 49% for scheme 1 (*p* < 0.05), 75% for scheme 2 (*p* < 0.0001), 80% for scheme 3 (*p* < 0.001), and 100% for scheme 4 (*p* < 0.0001). Schemes 2, 3, and 4 showed a feature of febrile neutropenia (counts < 0.5 × 10^9^/L). However, null or non-detectable neutrophil counts obtained in scheme 4 may make it less attractive to assess the role of potential protective agents.

As the results revealed so far, scheme 3 joins together most of the characteristics desirable for the aimed experimental model. First, decreases in paw and tail pressure thresholds (two of the three nociceptive tests assessed) were observed, which validates and gives confidence to the model. Although scheme 4 showed similar features, the undetectable neutrophil counts are a severe outcome that would make it difficult to evaluate potential myeloprotective agents. Schemes 1 and 2 only showed responses in one of the nociceptive tests performed. The following results were obtained by applying scheme 3 as a model of simultaneous induction of peripheral neurotoxicity and myelotoxicity by docetaxel.

Table 1 shows the remaining hemogram data from rats in scheme 3. The DT group had decreased counts in white blood cells (WBCs, leukopenia) and red blood cells (RBCs, erythropenia) compared to the CT group. Indeed, lower counts were observed in all blood cells except for basophils (BAS). In addition, hemoglobin (HGB) levels were also lowered by docetaxel administration. Additional tests were performed on rat blood plasma to determine other types of toxicity than neuro- and myelotoxicity. As shown in Table 1, plasma creatinine, alkaline phosphatase (ALP), aspartate aminotransferase (AST), alanine aminotransferase (ALT), and creatine kinase MB (CK-MB, mainly found in cardiac muscle) isoenzyme activity determinations produced lower levels in the DT group related to the non-treated group, with creatinine and CK-MB displaying values within normal physiological ranges.

### 2.2. Protective Role of Dimethyl Fumarate Against Docetaxel-Induced Nociceptive Alterations and Neutropenia

As seen above, animals of the DT group experienced a significant decrease in body weight gain throughout the treatments (Figure 1). This trend was neither counteracted nor attenuated in the group co-treated with dimethyl fumarate (100 mg/kg/week) and docetaxel (DT+DF group, Supplementary Figure S1a). Body weight gain in the dimethyl fumarate-treated group (DF) was similar to that for the CT group. Body weight in both docetaxel and co-administered groups showed significant differences since the second dose compared to the CT group (*p* < 0.05, Supplementary Figure S1a).

#### 2.2.1. Nociceptive Alterations and Neutropenia

**Figure 4:**
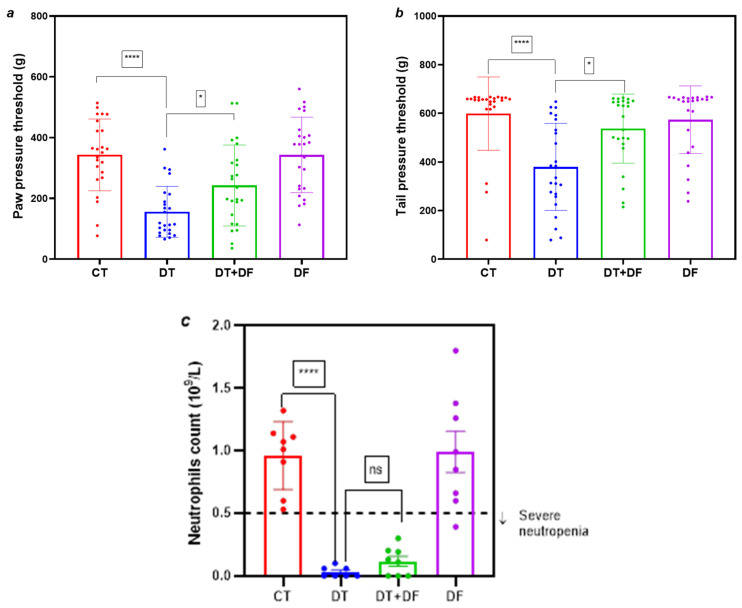
Effect of dimethyl fumarate on docetaxel-induced nociceptive alterations and neutropenia in Wistar rats. Values for pressure thresholds in paw (**a**) and tail (**b**) are given in grams (g), and values of neutropenia (**c**) are given in number of neutrophils ×10^9^/L. CT: control group; DT: docetaxel group; DT+DF: docetaxel + dimethyl fumarate group; DF: dimethyl fumarate group. Data are expressed as mean ± SD and analyzed with one-way analysis of variance (ANOVA) followed by Bonferroni multiple comparison tests (# Neutrophils) or Kruskal–Wallis test (paw and tail pressure), n = 8. **** *p* < 0.0001 vs. CT; * *p* < 0.05 vs. DT; ns: not significant.

Co-treatment with a single dose of dimethyl fumarate in the DT+DF group attenuated docetaxel-induced decreases in paw (Figure 4a, *p* < 0.05 vs. DT) and tail (Figure 4b, *p* < 0.05 vs. DT) pressure nociceptive thresholds. Paw and tail pressure thresholds due to docetaxel administration fall to approximately 45% and 63% of the CT value, respectively. Such nociceptive attenuations were determined as the paw and tail pressure thresholds in the DT+DF rats reached 71% and 90% of the CT group mean value, respectively. These values in the DF group were similar to those for the CT group.

Although some individual neutrophil counts were higher than those in the DT group, there was no significant neutropenia amelioration in the DT+DF group. Treatment with only dimethyl fumarate (DF group) did not affect neutrophil count compared to the CT group (Figure 4c).

#### 2.2.2. Histopathological Examination

**Figure 5:**
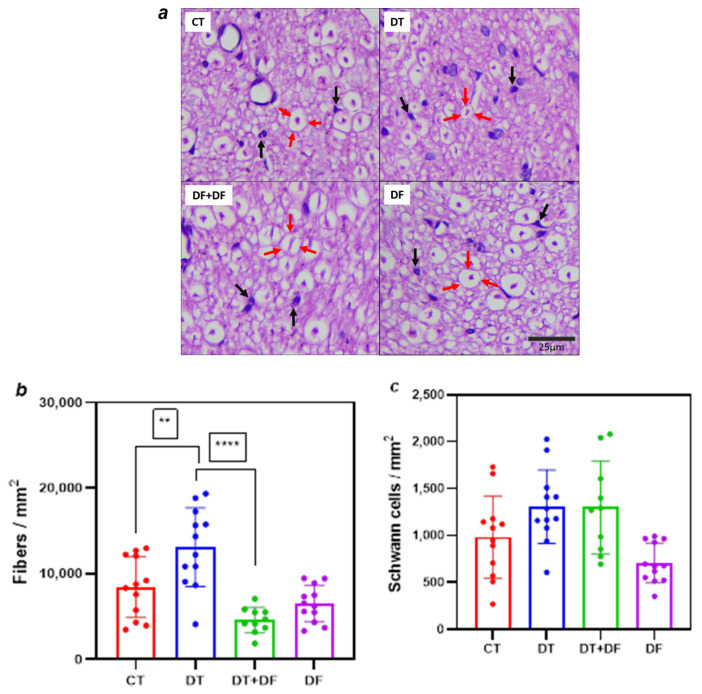
Effect of dimethyl fumarate on docetaxel-induced damage in nerve fibers of rat sciatic nerves. (**a**) Representative images (40×) of histological transverse sections of sciatic nerves of the rats under study stained with H&E. In the CT group, nerve fibers (rounded by red arrows) are arranged compactly, exhibiting a relatively homogeneous size. An increase in fiber density is observed in the DT group compared to the CT group, with more interstitial spaces and smaller fibers, suggesting a potential degenerative process. In the DT+DF and DF groups, fiber size distribution and density are similar to those of the CT group. Schwann cell (black arrows) density appears unchanged among all groups. (**b**) Nerve fiber density quantification. (**c**) Schwann cell density quantification. CT: control group; DT: docetaxel group; DT+DF: co-administered group; DF: dimethyl fumarate group. Data are expressed as mean ± SD and analyzed with one-way ANOVA followed by Bonferroni multiple comparison tests, n = 8. ** *p* < 0.02 vs. CT; **** *p* < 0.0001 vs. DT.

Figure 5a shows histological sections of sciatic nerves stained with hematoxylin and eosin (H&E). Nerve fibers in the CT group are arranged compactly, exhibiting uniform density and relatively homogeneous size (nerve fiber with its myelin sheath space and axoplasm are indicated by red arrows in Figure 5a). Conversely, in the DT group, fiber density is increased (Figure 5b, *p* = 0.0034 vs. CT), accompanied by numerous interstitial spaces and the presence of smaller fibers, suggesting a potential degenerative process (Figure 5a). In the co-treatment scheme (DT+DF group), fiber size distribution and density are similar to those of the CT group, with some fibers displaying increased diameters and a moderate decrease in overall density related to the DT group (Figure 5b, *p* < 0.0001). The DF group maintains a fiber density comparable to that of the CT group, with well-defined fibers of uniform size. On the other hand, Schwann cell density (black arrows in Figure 5a) did not change significantly throughout the experimental groups; however, a slight increasing trend in DT and DT+DF tissues, compared to the CT and DF groups (Figure 5c), can be seen.

Additionally, fascicle area, fibroblast density, and the nerve fiber/Schwann cell ratio were analyzed (Table 2). The S100 immunostain, Masson’s trichrome staining, and leukocyte common antigen (LCA) tests were also performed to identify Schwann cells, collagen fibers from extracellular matrix, and leukocyte activity, respectively, in transverse histological sections of sciatic nerves from the DT group (Supplementary Figure S2). This examination led to disregarding a likely inflammation process mediating the observed peripheral neuropathy induced by docetaxel.

**Figure 6:**
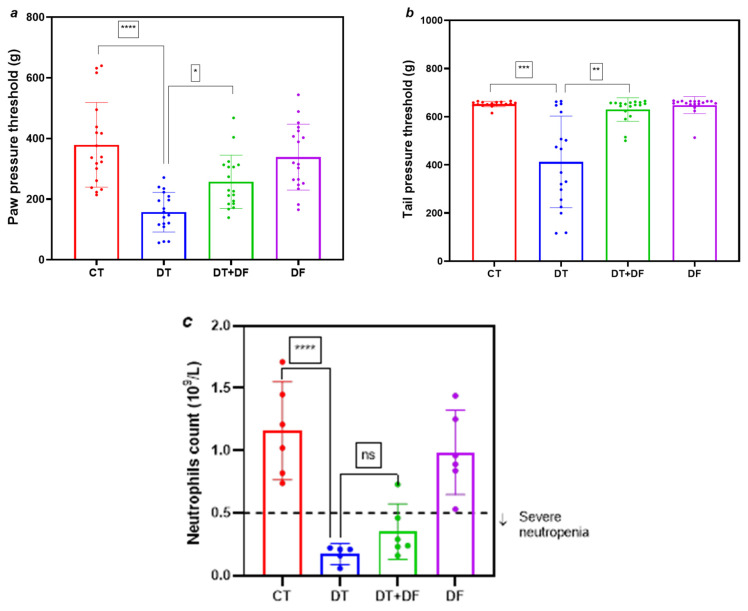
Effect of dimethyl fumarate administered as pre- and co-treatment (second dosage) on docetaxel-induced nociceptive alterations and neutropenia in Wistar rats. Values for pressure thresholds in paw (**a**) and tail (**b**) are given in grams (g), and values of neutropenia (**c**) are given in number of neutrophils ×10^9^/L. CT: control group; DT: docetaxel group; DT+DF: co-administered group; DF: dimethyl fumarate group. Data are expressed as mean ± SD and analyzed with one-way ANOVA followed by Bonferroni multiple comparison tests (# Neutrophils) or Kruskal–Wallis test (paw and tail pressure thresholds), n = 6. *** *p* < 0.001, **** *p* < 0.0001 vs. CT; * *p* < 0.05, ** *p* < 0.02 vs. DT, ns: not significant.

In addition to the same-day co-administration scheme of dimethyl fumarate and docetaxel, a scheme with a different dimethyl fumarate dosage was tested, aiming to improve the effect on nociception and neutropenia. This dosage consisted of four daily doses of dimethyl fumarate (100 mg/kg) before docetaxel administration and a further one administered on the same day as docetaxel. Treatment this way did not improve body weight gain compared to the first dimethyl fumarate dosage (Supplementary Figure S1). Paw and tail pressure thresholds also appeared similar between the DT+DF groups of both dosage regimens (Figure 4a,b and Figure 6a,b).

Regarding docetaxel-induced neutropenia, for this second dimethyl fumarate dosage, a trend to improve the neutrophil count in the DT+DF group compared to the first dosage was found (Figure 4c and Figure 6c, *p* < 0.02), while the neutrophil count in the DT+DF group of the first dimethyl fumarate dosage reached approximately 12% of that for the CT group (Figure 4c). This value was approximately 35% in the second dosage regimen (Figure 6c). However, no significant attenuation of neutropenia was observed in any case.

**Figure 7:**
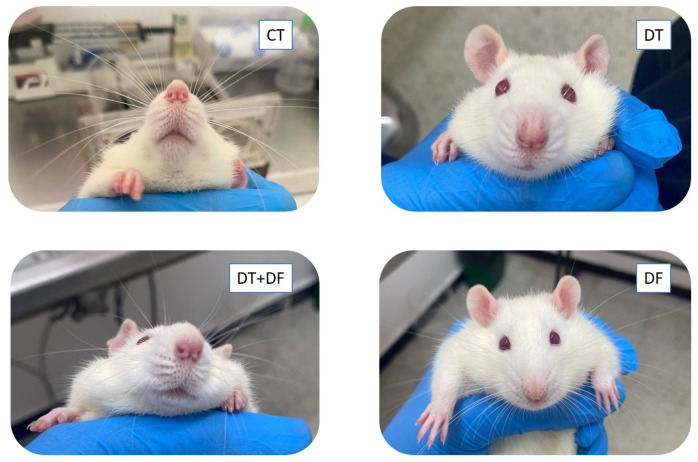
Abundance of vibrissae in four rats at the end of treatment with docetaxel and dimethyl fumarate (second dosage). CT: control rat; DT: rat treated with docetaxel; DT+DF: rat treated with docetaxel + dimethyl fumarate; DF: rat treated with dimethyl fumarate. n = 1.

A serendipitous finding registered with only four of the last rats under study suggests another type of docetaxel toxicity phenotype assessed by this experimental model. Images in Figure 7 show a DT rat with decreased vibrissae length and abundance compared to a control rat (CT). Strikingly, the rat belonging to the DT+DF group (second dimethyl fumarate dosage) seems to have attenuated this docetaxel-induced loss of vibrissae. The rat treated only with dimethyl fumarate (DF) looked similar to the control. Unfortunately, this effect could not be quantified because of an insufficient number of animals at the time, so a new study is required to validate these observations.

### 2.3. Effect of Dimethyl Fumarate and Docetaxel Combinations in Prostate Cancer Cell Lines

Lethal concentration 50 (LC_50_) of dimethyl fumarate or docetaxel was determined for both PC3 and LNCaP prostate carcinoma cell lines from the dose–effect curve of each individual treatment (Supplementary Figures S3 and S4). The LC_50_ of docetaxel was 170.94 nM for PC3 cells and 61.23 nM for LNCaP cells, while the LC_50_ of dimethyl fumarate was 14.61 μM for PC3 cells and 32.98 μM for LNCaP cells. Based on these concentrations, a combination scheme was designed using concentrations equivalent to one-fold LC_50_ (1 × LC_50_) or half of LC_50_ (0.5 × LC_50_) at the docetaxel-to-dimethyl fumarate (DT:DF) ratios 5:1, 2:1, 1:1, 1:2, and 1:5.

For all combinations evaluated in PC3 cells, the Chou-Talalay classification was applied (Table 3). Synergistic effects were observed at most ratios evaluated, with the 5:1 ratio showing a “very strong synergy” as the combination index (CI_X_) gave values < 0.1. At equipotent ratios (1:1), the analysis revealed “synergism” when concentrations equivalent to 1 × LC_50_ were used and “strong synergism” at 0.5 × LC_50_. Conversely, combinations with 1:5 ratios showed interactions “nearly additive” and “antagonism” (CI_X_ ranged between 0.90–1.10 and 1.45–3.3, respectively).

Combinations in LNCaP cells showed diverse effects (Table 4), ranging from “strong synergism” at the 5:1 and 1:1 (0.5 × LC_50_ concentrations) ratios to “nearly additive” and “lack of effect” in 1:2 and 1:5 ratios, respectively. The description “lack of effect” refers to a combination in which the response did not significantly differ from the untreated control, and the calculated CI_X_ value was out of range, indicative of antagonism.

## 3. Discussion

Due to its toxicity profile, docetaxel treatment is frequently associated with 24–35% of patients requiring infusion delays, 12–22% requiring dose reductions, and 11–20% discontinuing therapy altogether [27]. Nevertheless, docetaxel remains a widely used standard agent for improving overall survival in advanced cancers [27]. These challenges urge the development of strategies to mitigate adverse effects and widen the therapeutic index of this chemotherapeutic.

As anticipated, docetaxel administration led to general toxicity, initially evidenced by the weight loss in the DT group across all treatment schemes (Figure 1). This finding aligns with decreased feeding behavior, previously reported in both preclinical and clinical studies [28,29,30]. The reduced levels of HGB, creatinine, and plasma enzymes—ALP, AST, ALT, and CK-MB—observed in scheme 3 (Table 1) may be attributed to this nutritional deficiency. Like us, Zhao et al. [28] also reported decreases in ALP, AST, and ALT, contrasting with other studies where chemotherapeutic agents typically elevated these enzymes [31]. Additionally, docetaxel has been linked to capillary protein leak syndrome, reducing colloid osmotic pressure and potentially increasing plasma volume, which could further explain the systematic reduction in plasma markers observed in the DT group of scheme 3 [32].

Nociceptive alterations induced by docetaxel have previously been explored in rats [29], though not concurrently with markers of myelotoxicity. Increases in nociceptive thresholds observed in scheme 1 (paw pressure, Figure 2a) and scheme 4 (hot plate, Figure 2c) were not considered for further analysis, as such elevations are not canonical outcomes in preclinical peripheral neuropathy assessments. The hot plate test showed no significant differences in most schemes, making mechanical nociceptive stimuli reliable for this model rather than thermal stimuli. Schemes 3 and 4 showed significant decreases in paw and tail pressure thresholds (Figure 2a,b), indicative of mechanical hyperalgesia in rodents. These findings support the presence of peripheral neuropathy, defined by enhanced nociceptive responses to potentially tissue-damaging stimuli [33].

Neutropenia, a well-established side effect of docetaxel [34], has been effectively modeled in rodents [16,35]. All schemes tested in this study showed significantly reduced neutrophil counts relative to controls (Figure 3). However, only schemes 2–4 reached neutrophil levels consistent with febrile neutropenia (<0.5 × 10^9^/L). Scheme 4 was excluded from further analysis due to undetectable neutrophil counts, which complicates the evaluation of potential protective agents against myelotoxicity. Based on pain behavior and hematological outcomes, scheme 3 emerged as the most appropriate model for the concurrent assessment of peripheral neuropathy and neutropenia. Furthermore, scheme 3 recapitulated other docetaxel-associated hematological alterations seen in clinical practice, such as leukopenia, anemia, and lymphopenia [30], as well as less frequently reported changes such as monocytopenia and eosinopenia (Table 1).

Employing scheme 3, we evaluated the dual neuro- and myeloprotective potential of dimethyl fumarate in the context of docetaxel-induced toxicity. A single 100 mg/kg dose of dimethyl fumarate co-administered with docetaxel mitigated mechanical hyperalgesia, suggesting a protective role in peripheral nerves (Figure 4). Previous studies support this result, showing that dimethyl fumarate confers neuroprotection in models of surgically induced neuropathy [18] and in chemotherapy-induced neuropathies triggered by agents such as oxaliplatin [36] and paclitaxel [21]. Pre-treatment with four doses of dimethyl fumarate elicited similar outcomes against hyperalgesia (Figure 6a,b). While significant protection against neutropenia was not observed, a positive trend in neutrophil recovery was noted with increased dimethyl fumarate dosing (Figure 6c), warranting further investigation into larger-dose regimens or a higher number of doses before docetaxel.

Histological analysis of sciatic nerve sections revealed increased nerve fiber density in the DT group compared to controls, along with a trend toward elevated Schwann cell density that persisted in the DT+DF group (Figure 5, Table 2). Similar patterns were described by Persohn et al. [37], who found an increased density of myelinated fibers in paclitaxel-treated rats, accompanied by decreased axonal diameter and a shift toward smaller-caliber fibers. Although both paclitaxel and docetaxel significantly reduced nerve conduction velocity, the severity of histopathological changes was milder than expected. The observed increase in axonal density, likely due to decreased caliber without fiber loss, suggests compensatory neuroplastic changes associated with taxane-induced neuropathy. Such structural alterations may stem from microtubule dysfunction and impaired axonal transport, affecting the neuronal cytoskeleton [37]. Interestingly, the DT+DF group exhibited fiber densities comparable to the control group, further supporting a neuroprotective effect.

As a preliminary finding, the protective effect of dimethyl fumarate against docetaxel-induced mechanical hyperalgesia determined in our study could be attributed to the Nrf2-mediated antioxidant capacity of dimethyl fumarate reported before. We mentioned above the work by Singh et al., in which Nrf2 expression is increased in sciatic nerves of rats co-treated with paclitaxel, a docetaxel analog, and dimethyl fumarate, with the consequent amelioration of neuropathic pain [21]. Previously, Kawashiri et al. [38] demonstrated the dimethyl fumarate-associated nuclear DNA binding of Nrf2 in the neuron cell-like line PC12, thereby preventing the neurite damage (a marker of neurodegeneration) induced by oxaliplatin. Besides its antioxidant cell response via Nrf2, dimethyl fumarate has also been linked to preservation of mitochondrial functions in diabetic neuropathic rats, as was reported by Jindam et al. [39]. Unlike these works reporting the role of Nrf2 as a mediator of neuroprotection, N-acetylcysteine, another well-known antioxidant and Nrf2 inducer, failed to prevent and control taxane-induced peripheral neuropathy in patients with breast cancer enrolled in a randomized triple-blind clinical trial [40]. However, the authors of this study justified this lack of protection by the poor oral bioavailability of N-acetylcysteine [40]. Other works highlighting the role of Nrf2 modulated by distinct inducers (e.g., resveratrol, curcumin, and sulforaphane) on experimental models of neuropathic pain were reviewed by Petrikonis et al. [41]. Most of the mechanisms described in this review imply the activation of Nrf2 to reduce oxidative damage, inflammation, and mitochondrial impairment. In the present work, we disregarded an inflammatory process in nerve fibers, as was indicated above (Supplementary Figure S2). Currently, we are setting up experiments to explore the effect of dimethyl fumarate on Nrf2 nuclear translocation and mitochondrial function in the sciatic nerve, specifically the axonal transport of mitochondria, which is meant to be altered in the docetaxel-induced peripheral neuropathy [42].

The rat model established here also allowed for observing vibrissae loss due to docetaxel exposure (Figure 7), possibly mirroring chemotherapy-induced alopecia observed in up to 83.3% of patients receiving docetaxel [6]. Although strategies such as scalp cooling have been employed in patients receiving docetaxel chemotherapy, alopecia remains a persistent adverse effect [43]. Co-treatment with dimethyl fumarate appeared to reduce vibrissae loss in rats (DT+DF group, Figure 7). This aligns with findings by Haslam et al. [44], who demonstrated that Nrf2 activation alleviates oxidative stress in human hair follicles, suggesting a plausible protective strategy against chemotherapy-induced hair damage. Given the anatomical and physiological similarities between rodent vibrissae and human scalp follicles, this model may be suitable for further investigations into the protective potential of Nrf2 activators such as dimethyl fumarate against chemotherapy-induced alopecia.

Our results in PC3 and LNCaP cell cultures (Table 3 and Table 4 and Supplementary Figures S3 and S4) suggest that the neuroprotective effect of dimethyl fumarate in vivo would not compromise the antitumoral efficacy of docetaxel. On the contrary, most of the combination ratios evaluated showed a synergistic effect, particularly the equipotent ratios (DT:DF ratio 1:1) and those in which the potency of docetaxel is higher than that of dimethyl fumarate (DT:DF ratios 5:1 and 2:1). As a concluding remark of these outcomes, avoiding combination ratios in which the dimethyl fumarate potency is higher (e.g., DT:DF ratios 1:2 and 1:5 in this study) is critical. Pharmacokinetic assessments in rats exposed to docetaxel plus dimethyl fumarate combinations will be important to confirm that the synergistic concentration ratios found in cell culture can be obtained in rat plasma and tissues. A murine prostate cancer xenograft model under a combination scheme of docetaxel plus dimethyl fumarate would allow us to validate the outcomes generated so far in the same animal, antitumoral synergism and protection against peripheral neuropathy.

## 4. Materials and Methods

### 4.1. Drugs

Docetaxel was purchased from BLAŪ Farmacéutica (Cotia, SP, Brazil), and prepared and mixed according to the manufacturer’s instructions. Dimethyl fumarate (97%) was purchased from Sigma-Aldrich (St. Louis, MO, USA) and resuspended in carboxymethyl cellulose (CMC, Sigma-Aldrich), which was prepared at 1% *w*/*v* by diluting in distilled water.

### 4.2. Animals

Male Wistar rats weighing 240 ± 30 g at the start of the treatments were used. Animals were maintained, cared for, and used following the Guide for the Care and Use of Laboratory Animals of the National Academy of Sciences of the United States [45]. Animals were kept in stainless steel cages 30 cm × 40 cm × 20 cm high (6 rats per cage) at a temperature of 21–24 °C, relative humidity of 55–70%, photoperiod of 12 h of light and 12 h of darkness with sterile water and specialized food ad libitum. The diet consisted of a 1:1 mixture of two commercial maintenance feeds. The resulting nutritional profile of this blend included a minimum crude protein content of 18–19%, minimum crude fat of 5–6%, maximum crude fiber of 6–7%, maximum moisture content of 12%, and a maximum ash content of approximately 9%. Protocols were approved by the Ethics Committee in Scientific Research (CEINCI) of the Universidad Industrial de Santander (Committee minutes No. 10 of 2021 and No. 12 of 2024).

Rats were habituated and trained daily in the morning to diminish stress levels and improve the model reliability. Four days before starting the administration schemes, rats were habituated to the room, caretakers, researchers, and equipment used in experiments. Animals also underwent individual training to teach them to remain still and calm during oral administration, restraining for intravenous (i.v.) administration, and using analgesimeters.

### 4.3. Establishment of the Docetaxel Damage Model

**Figure 8:**
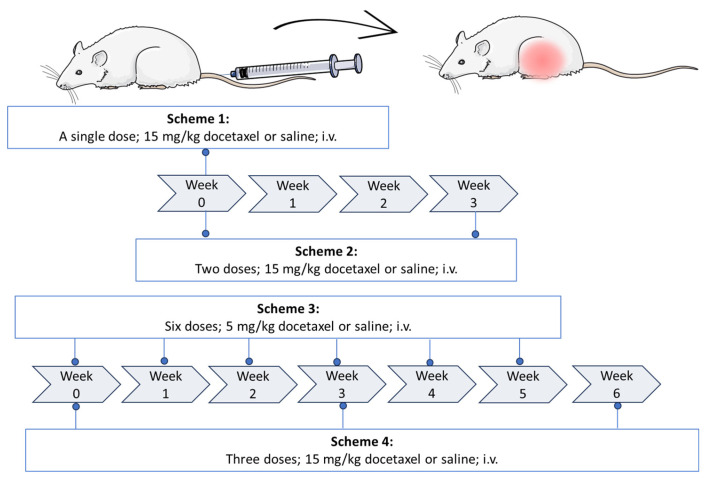
Docetaxel administration schemes assessed for simultaneous induction of peripheral neuropathy and neutropenia. i.v., intravenously.

Four docetaxel administration schemes were tested, varying dose and dosage (Figure 8). Scheme 1 consisted of a 15 mg/kg single dose. Scheme 2 included two doses of 15 mg/kg administered three weeks apart. In scheme 3, a 5 mg/kg dose was administered weekly until 6 doses were completed. Scheme 4 consisted of three 15 mg/kg doses, each administered every 3 weeks. In all cases, docetaxel was administered i.v. in the tail. Each scheme evaluated two experimental groups, the control group (CT, n = 8), which was administered isotonic saline solution, and the docetaxel-treated group (DT, n = 8). Administration schemes were proposed based on clinical practice and trials from the literature [46,47], equivalent for rats [48]. However, some reports where either peripheral neuropathy or neutropenia was induced in rats, but not simultaneously, were also considered [29,35]. Group sizes were obtained using the “resource equation” method, due to the limited and variable information on effect size and standard deviation of the results [49]. According to the resource equation, the appropriate sample size to use in this research was in the range of 6 to 11 subjects. A sample size of n = 8 was used for the implementation of the damage model with docetaxel.

### 4.4. Behavioral Testing, Euthanasia, and Blood Cell Count

Four days after the last administration in each scheme, sensory behavioral testing, paw and tail pressure, and hot plate were performed using the PANLAB analgesimeter model LE7306 Randall-Selitto and the PANLAB analgesimeter model LE7406 Hot Plate (Panlab, S.L.U., Barcelona, Spain), respectively. Nociceptive testing was based on physical indicators of discomfort (withdrawal reflexes, licking, and voicing). To ensure ethical treatment of the animals, maximum cut-off values of 10 s of paw and tail pressure latency and a weight of up to 650 g were adjusted. For the case of the hot plate method, 60 s of latency at 50 ± 0.1 °C was taken as the cut-off. Once the nociceptive tests were concluded, the rats were euthanized under anesthesia with ketamine (90 mg/kg) + xylazine (15 mg/kg) intraperitoneally (i.p.) by exsanguination through the left ventricle of the animal.

### 4.5. Hemograms and Blood Biochemistry

Complete differential cell counts were performed in blood samples to determine neutropenia using a Genvet VH50 hematology analyzer (Genrui Biotech Inc., Shenzhen, China). Blood biochemistry tests were performed using kits commercially available following the manufacturer’s instructions (LabTest Diagnóstica S.A., Minas Gerais, Brazil); creatine kinase activity (CK-MB. Ref. 118), aspartate aminotransferase (AST, Ref. 109), alanine aminotransferase (ALT, Ref. 1008), alkaline phosphatase (Ref. 79), creatinine K concentration (Ref. 96), and urea (Ref. 104) were analyzed using the Varioskan LUX multimode microplate reader (Thermo Fisher Scientific, Waltham, MA, USA).

### 4.6. Assessment of Dimethyl Fumarate Protection

Once selected, the best scheme that ensured neutropenia and peripheral neurotoxicity was challenged with dimethyl fumarate. This administration scheme consisted of a control group (CT, n = 8), which received saline i.v. as a vehicle for docetaxel and oral CMC as a vehicle for dimethyl fumarate; a docetaxel group (DT, n = 8), administered with docetaxel i.v. along with CMC; a co-treated group (DT+DF, n = 8) administered with docetaxel i.v. and dimethyl fumarate orally at 100 mg/kg/day [50,51]; an additional control group was administered with only dimethyl fumarate (DF, n = 8) and saline as vehicle for docetaxel. Docetaxel or its vehicle was administered according to the scheme selected for the simultaneous induction of nociceptive alterations and neutropenia. Dimethyl fumarate or CMC was administered 1 h before docetaxel (or its vehicle). A second dimethyl fumarate dosage was tested in which dimethyl fumarate or CMC was administered daily, four days before and on the same day as docetaxel or its vehicle (n = 6). A sample size of n = 8 was used in the first dimethyl fumarate dosage, and n = 6 in the second dosage. A representation of both dosages of dimethyl fumarate is given in the Supplementary Figure S5.

As previously described for the establishment of the damage model, four days after the end of the treatment, animals were subjected to sensory behavioral tests and subsequently euthanized, obtaining blood for hemograms and blood biochemistry. In addition, sciatic nerves were removed and stored for further studies on the mechanisms of damage and protection.

### 4.7. Histopathology

After completing the treatments and co-treatments, the rats were euthanized and perfused with phosphate-buffered saline (PBS) to wash the organs, followed by fixation with a 10% formalin solution (Sigma-Aldrich). Both sciatic nerves were then excised and preserved in 10% formalin. Tissues were processed for paraffin embedding and sectioned using a Leica RM 2255 microtome (Leica Biosystems, Deer Park, IL, USA) at 4 µm thickness.

Sections were stained with H&E (J.T.^®^, Center Valley, PA, USA) and Masson’s trichrome stain (Sigma-Aldrich). S100 immunohistochemical analysis to identify Schwann cells and LCA immunohistochemical analysis to identify leukocytes were performed using rabbit polyclonal anti-S100 antibody (Abcam, Cambridge, UK) and mouse monoclonal anti-CD45 antibody (Abcam), respectively. Images were captured using an Olympus BX51 microscope (Olympus Corporation, Center Valley, PA, USA), and morphometric measurements were performed with image processing software Image-Pro^®^Plus version 6.0 (Media Cybernetics, Rockville, MD, USA). Five fields were randomly selected for each section at 40× magnification.

### 4.8. Cell Culture

PC3 prostate carcinoma (ATCC^®^—CRL-1435) and LNCaP prostate carcinoma (clone FGC, ATCC^®^—CRL-1740) were obtained from the American Type Culture Collection (Rockville, MD, USA). Cell lines were grown in RPMI 1640 (Sigma Chemicals Aldrich, Milan, Italy) supplemented with 2% fetal bovine serum (Gibco, Thermo Fisher Scientific, Waltham, MA, USA), 1% penicillin-streptomycin solution (Sigma-Aldrich), and 1% GlutaMAX™ Supplement (Gibco™, Thermo Fisher Scientific). For cytoxicity assay, cells were seeded in 96-well culture plates (Corning, New York, NY, USA) at a density of 15,000 cells per well and were allowed to attach and recover for at least 24 h. Dose-effect curves were prepared by incubating cells with culture medium containing dimethyl fumarate or docetaxel at different concentrations for 72 h. Cell viability was normalized to an untreated control and a dead control treated with 100 mM trisodium citrate (PanReac AppliChem, Darmstadt, Germany) in culture medium RPMI 1640.

### 4.9. Crystal Violet Assay

The crystal violet assay was performed to measure the cytotoxic effect of docetaxel, dimethyl fumarate, and their combinations on PC3 and LNCaP cells according to Negrette-Guzmán et al. [52]. Briefly, cells were washed with 100 µL of 1X PBS per well and fixed with 100 µL of 4% paraformaldehyde (Sigma-Aldrich) in PBS for 30 min. Then, 50 µL of 0.2% crystal violet (Scharlau, Scharlab S.L., Sentmenat, Spain) in 6% methanol (Supelco, Merck, Darmstadt, Germany) was added and incubated at room temperature for 30 min. Excess dye was removed by washing with distilled water, and 100 µL of 0.5% sodium dodecyl sulfate (J.T. Baker) in 0.1 M citrate with 50% ethanol (PanReac AppliChem) was added to incubate at 37 °C with 5% CO_2_ for 1 h. Finally, absorbance was measured at 570 nm using the Varioskan LUX multimode microplate reader (Thermo Fisher Scientific).

### 4.10. Drug Combination Schemes

Docetaxel (DT) and dimethyl fumarate (DF) were administered either alone or in combination. For combination treatments, the drugs were added simultaneously at fixed DT:DF ratios (5:1, 2:1, 1:1, 1:2, and 1:5), using concentrations corresponding to 1 × IC_50_ and 0.5 × IC_50_ of each compound (Table 5). Drug interactions were analyzed using the median-effect analysis described by Chou-Talalay [53,54]. The CompuSyn software (version 1.0; ComboSyn, Inc., Paramus, NJ, USA) was used to calculate the combination index (CI_X_), where CI_X_ = 1 indicates an additive effect, CI_X_ < 1 indicates synergy, and CI_X_ > 1 indicates antagonism.

### 4.11. Statistical Analysis

All results were expressed as the mean ± SD of data. Data processing was performed by *t*-test (parametric test) or Mann–Whitney test (non-parametric test) analysis comparing groups DT and CT for each of the four evaluated damage schemes. Normal distribution of the data was determined using the Shapiro–Wilk test. To compare between groups in the protection schemes, ANOVA followed by Bonferroni multiple comparison test (parametric test) or Kruskal–Wallis test (non-parametric test) was used. Significance was determined at *p* ≤ 0.05. All data were processed, plotted, and analyzed using Prism (version 10.3.1, GraphPad Software, Boston, MA, USA).

## 5. Conclusions

An experimental rat model for the simultaneous induction of peripheral neuropathy and myelotoxicity by docetaxel, a clinically meaningful cancer drug, was established. The model is highly reliable and allows the evaluation of promissory protective agents against both adverse effects using fewer animals; it could even be used as a docetaxel-induced hair follicle damage model after some methodological validations.

Preliminarily, dimethyl fumarate was assessed as a therapeutic alternative against docetaxel toxicity, displaying amelioration of nociceptive and histological alterations. On the other hand, it showed no significant effect against docetaxel-induced neutropenia. However, the results suggest that more days in pre-treatment mode, larger doses of dimethyl fumarate, or both, may lead to significant protection against both adverse effects.

## Figures and Tables

**Table 1:** Complete blood count and other toxicity plasma markers from rats under scheme 3.

Scheme 3							
Test	Unit	Group					Normal Values
		CT		DT			
		Mean (95% CI)	SD	Mean (95% CI)	SD	*p* vs. CT	
WBC	10^9^/L	4.809 (3.586–6.031)	1.463	1.164 (0.892–1.435)	0.324	<0.0001	1.96–8.25
#LYM	10^9^/L	3.420 (2.422–4.418)	1.194	0.591 (0.051–1.131)	0.646	<0.0001	1.41–7.11
#MON	10^9^/L	0.313 (0.359–0.389)	0.110	0.026 (−0.003–0.055)	0.035	<0.0001	0.03–0.18
#EOS	10^9^/L	0.029 (0.018–0.039)	0.012	0.006 (0–0.012)	0.007	0.0047	0.01–0.16
#BAS	10^9^/L	0.008 (0.001–0.013)	0.007	0.004 (−0.0005–0.008)	0.005	>0.9999	0–0.005
RBC	10^12^/L	7.893 (7.620–8.165)	0.326	5.191 (4.821–5.563)	0.443	<0.0001	7.27–9.65
HGB	g/dL	16.313 (15.890–16.740)	0.511	11.888 (11.100–12.670)	0.942	<0.0001	13.7–17.6
Crea	mg/dL	0.733 (0.589–0.877)	0.142	0.568 (0.524–0.615)	0.041	0.0247	0.46–0.88
ALP	U/L	78.670 (58.480–98.870)	19.512	28.711 (25.710–31.710)	2.669	0.0002	85.4–311.7
AST	U/L	75.613 (55.070–96.160)	18.870	50.940 (41.130–60.740)	9.319	0.0416	65.8–266.2
ALT	U/L	29.682 (23.170–36.190)	6.569	19.337 (15.400–36.190)	3928	0.0165	26.3–68.5
Urea	mg/dL	31.899 (24.150–39.800)	7.722	30.411 (24.020–36.800)	6.424	>0.9999	19.62–23.02
CK-MB	U/L	2473.161 (1643–3303)	815.850	1989.088 (1211–2216)	784.760	0.648	233–4367

ALP: alkaline phosphatase; ALT: alanine aminotransferase; AST: aspartate aminotransferase; #BAS: basophil count; CI: confidence interval; CK-MB: creatine kinase MB; Crea: creatinine; CT: control group; DT: docetaxel group; #EOS: eosinophil count; HGB: hemoglobin; #LYM: lymphocyte count; #MON: monocyte count; RBC: red blood cell count; WBC: white blood cell count. Data are expressed as mean ± SD and analyzed with *t*-test, n = 8. Normal values were obtained from [25,26].

**Table 2:** Summary of morphometric measurements obtained from histopathological analysis of sciatic nerve sections from rats co-treated with docetaxel and dimethyl fumarate.

Analysis	GROUP									
	CT		DT			DT+DF			DF	
	Mean (95% CI)	SD	Mean (95% CI)	SD	*p* vs. CT	Mean (95% CI)	SD	*p* vs. DT (95% CI)	Mean (95% CI)	SD
Fibre density/mm^2^	8433 (6191–10,675)	3529	13,062 (10,143–15,980)	4.593	0.0034	4557 (3500–5614)	1478	<0.0001	6465 (5122–7808)	2114
Fascicle area (μm^2^)	198,591 (−53,588–450,770)	301,642	51,480 (25,364–77,596)	41,104	0.2254	80,093 (43,776–116,411)	34,607	0.1969	61,787 (17,00–121,874)	57,257
Schwann cell density/mm^2^	984.0 (706.5–1261)	436.8	1306 (1059–1555)	390.4	0.2666	1300 (946.4–1654)	494.5	>0.9999	705.7 (571.3–840.1)	211.6
Fibroblast density/mm	25.00 (14.14–35.86)	8.746	26.75 (15.11–38.39)	13.93	>0.9999	26.00 (20.62–31.38)	6.437	>0.9999	25.00 (20.35–20.65)	3.742
Nerve fiber to Schwann cell ratio	9.167 (7.313–11.02)	2.918	10.00 (8.352–11.65)	2.594	0.9520	3.800 (2.921–4.679)	1.229	<0.0001	9.500 (7.819–11.18)	2.646

CI: confidence interval; CT: control group; DT: docetaxel group; DT+DF: docetaxel + dimethyl fumarate group; DF: dimethyl fumarate group. Data are expressed as mean ± SD and analyzed with one-way ANOVA followed by Bonferroni multiple comparison tests or Kruskal–Wallis test, n = 8.

**Table 3:** Chou-Talalay analysis of docetaxel:dimethyl fumarate (DT:DF) combinations in PC3 cells.

DT:DF Ratio	Group	DT [nM]	DF [μM]	Effect	CI_X_	95% CI	Range of CI_X_	Description
5:1	1 × CL_50_	171	2.9	0.84959	0.06572	(0.039–0.093)	<0.1	Very strong synergism
	0.5 × CL_50_	85.5	1.46	0.78708	0.05924	(0.035–0.083)	<0.1	Very strong synergism
2:1	1 × CL_50_	171	7.31	0.75420	0.29597	(0.173–0.419)	0.3–0.7	Synergism
	0.5 × CL_50_	85.5	3.66	0.72731	0.18913	(0.110–0.268)	0.1–0.3	Strong synergism
1:1	1 × CL_50_	171	14.6	0.80724	0.34583	(0.241–0.451)	0.3–0.7	Synergism
	0.5 × CL_50_	85.5	7.31	0.79732	0.20048	(0.102–0.299)	0.1–0.3	Strong synergism
1:2	1 × CL_50_	85.5	14.6	0.79298	0.34551	(0.294–0.397)	0.3–0.7	Synergism
	0.5 × CL_50_	42.8	7.31	0.60237	0.44769	(0.383–0.513)	0.3–0.7	Synergism
1:5	1 × CL_50_	34.2	14.6	0.61317	0.77660	(0.611–0.942)	0.90–1.10	Nearly additive
	0.5 × CL_50_	17.1	7.31	0.38281	1.63338	(0.199–3.068)	1.45–3.3	Antagonism

CI: confidence interval; CI_X_: combination index; DT: docetaxel; DF: dimethyl fumarate. CI_X_ was calculated with 95% CI. The highest CI_X_ (95% CI) value observed was adjusted according to the Chou-Talalay classification, n = 3.

**Table 4:** Chou-Talalay analysis of docetaxel:dimethyl fumarate (DT:DF) combinations in LNCaP cells.

DT:DF Ratio	Group	DT [nM]	DF [μM]	Effect	CI_X_	95% CI	Range of CI_X_	Description
5:1	1 × CL_50_	61.0	6.59	0.75665	0.09356	(0.073–0.114)	0.1–0.3	Strong synergism
	0.5 × CL_50_	30.5	3.29	0.67837	0.11546	(0.037–0.194)	0.1–0.3	Strong synergism
2:1	1 × CL_50_	61.0	11.0	0.73709	0.17877	(0.126–0.231)	0.1–0.3	Strong synergism
	0.5 × CL_50_	30.5	5.49	0.64662	0.47484	(0.023–0.926)	0.90–1.10	Nearly additive
1:1	1 × CL_50_	61.0	32.9	0.86485	0.23696	(0.154–0.320)	0.3–0.7	Synergism
	0.5 × CL_50_	30.5	16.5	0.81565	0.16043	(0.116–0.205)	0.1–0.3	Strong synergism
1:2	1 × CL_50_	30.5	32.9	0.64410	0.79257	(0.523–1.062)	0.90–1.10	Nearly additive
	0.5 × CL_50_	15.2	16.5	0.10335	217,217,810	NA	NA	Lack of effect
1:5	1 × CL_50_	12.2	32.9	0.33615	22.00306	NA	NA	Lack of effect
	0.5 × CL_50_	6.10	16.5	0.01	242,900,000	NA	NA	Lack of effect

CI: confidence interval; CI_X_: combination index; DT: docetaxel; DF: dimethyl fumarate; NA: not applicable due to lack of measurable effect. CI_X_ was calculated with 95% CI. The highest CI_X_ (95% CI) value observed was adjusted according to the Chou-Talalay classification, n = 3.

**Table 5:** Drug combination schemes based on fixed DT:DF ratios for PC3 and LNCaP cell lines. DT: a, DF: b.

Rate 5:1	Rate 2:1	Rate 1:1	Rate 1:2	Rate 1:5
1 CL_50_ a:b	1 CL_50_ a:b	1 CL_50_ a:b	1 CL_50_ a:b	1 CL_50_ a:b
0.5 CL_50_ a:b	0.5 CL_50_ a:b	0.5 CL_50_ a:b	0.5 CL_50_ a:b	0.5 CL_50_ a:b

## Data Availability

The original contributions presented in this study are included in the article/Supplementary Materials. Further inquiries can be directed to the corresponding author(s).

## Supplementary Materials

The supporting information can be downloaded at: https://www.mdpi.com/article/10.3390/ijms26125859/s1**.**
