# Supplementary material for: An Experimental Rat Model for Simultaneous Induction of Peripheral Neuropathy and Myelotoxicity by Docetaxel Administration: Evaluating the Protective Role of Dimethyl Fumarate
Source: Int J Mol Sci. 2025 Jun 19;26(12):5859. doi: 10.3390/ijms26125859 (PMC12193057; doi:10.3390/ijms26125859)
Supplement: Supplementary file 1 [file ijms-26-05859-s001.zip › ijms-3559380-supplementary.pdf]

Supplementary data

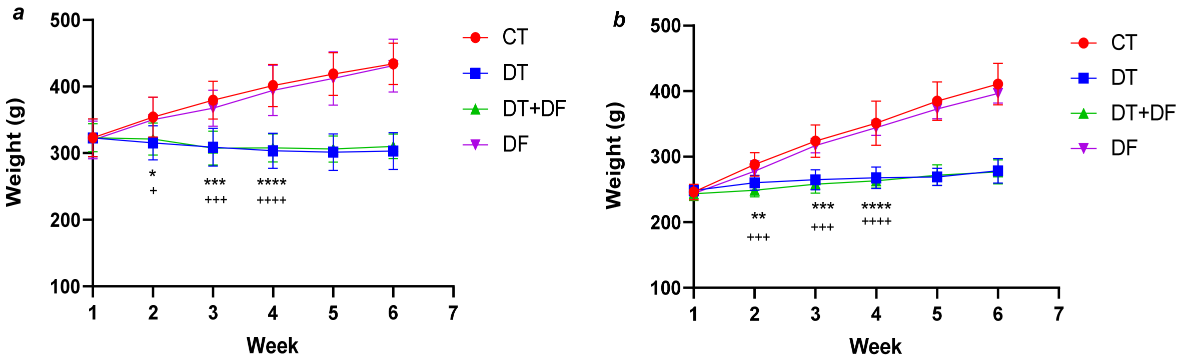

Supplementary Figure S1. Body weight gain in rats of a model for docetaxel-induced nociceptive alterations and neutropenia and evaluation of the dimethyl fumarate effect. **(a)** Co-treatment with dimethyl fumarate (100 mg/kg/week) and docetaxel (scheme 3) and **(b)** treatment with dimethyl fumarate (100 mg/kg/day) four days before and on the same day of treatment with docetaxel. Body weight was measured on the days of docetaxel and dimethyl fumarate administration. CT: control group; DT: docetaxel group; DT+DF: dimethyl fumarate + docetaxel group; and DF: dimethyl fumarate group. Data are expressed as mean ± SD and were analyzed by one-way ANOVA, using the Bonferroni test, n = 8. **$P < 0.02$, ***$P < 0.001$ DT vs CT; ++$P < 0.02$, ++++$P < 0.0001$ DT+DF vs CT.

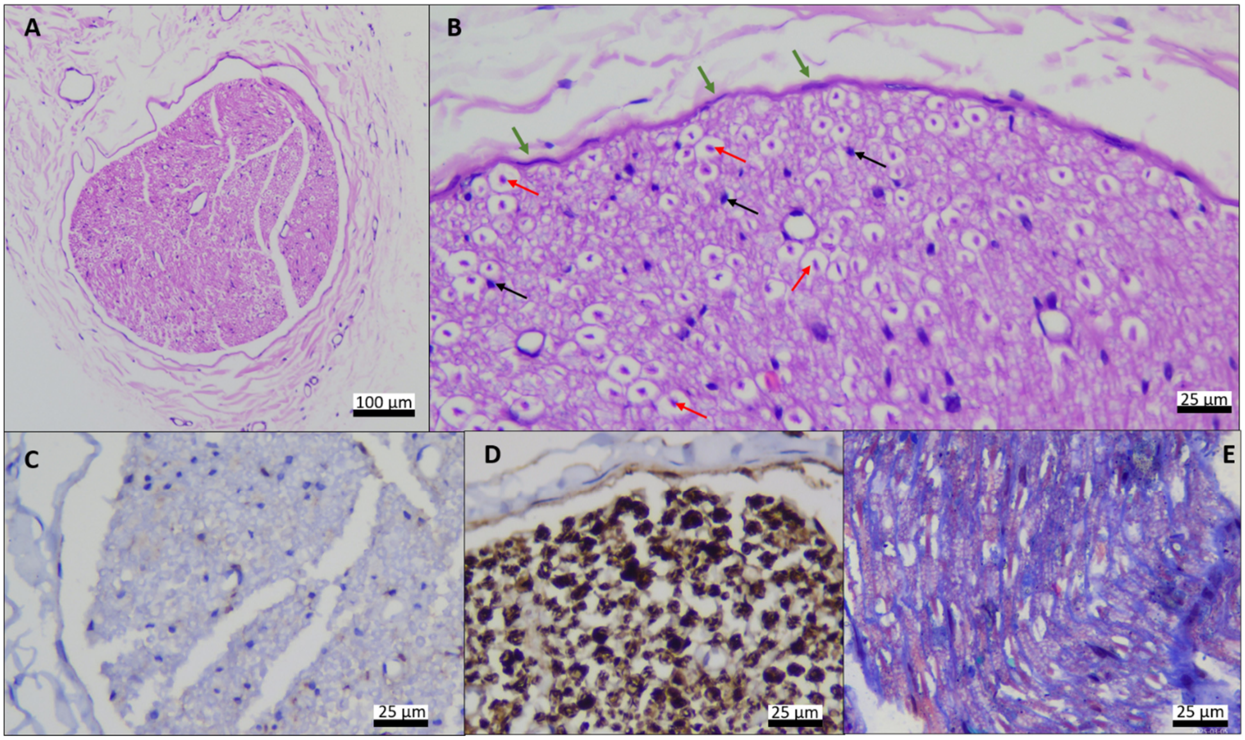

Supplementary Figure S2. Representative images of sciatic nerve transverse sections from rats treated with docetaxel (scheme 3) subjected to special staining techniques. **(A)** Transverse section of a nerve fascicle stained with H & E reveals the organization of nerve fibers within the endoneurium. **(B)** A high-magnification (40 X) image of the epineurium and perineurium illustrates the distribution of Schwann cells (black arrows) and the surrounding extracellular matrix. Within the fascicle, axons (red arrows) appear as clear areas due to the removal of myelin by solvents used in histological processing. The endoneurium consists of loose connective tissue interspersed with elongated nuclei belonging to Schwann cells and fibroblasts (green arrows). Wavy collagen fibers are visible at the periphery. **(C)** Immunohistochemical staining for LCA shows no expression or reactivity, indicating the absence of inflammatory cells in the tissue. **(D)** Immunohistochemical staining for the S100 protein highlights Schwann cell expression. **(E)** Masson's trichrome staining shows connective tissue support within the endoneurium, with collagen fibers appearing in blue.

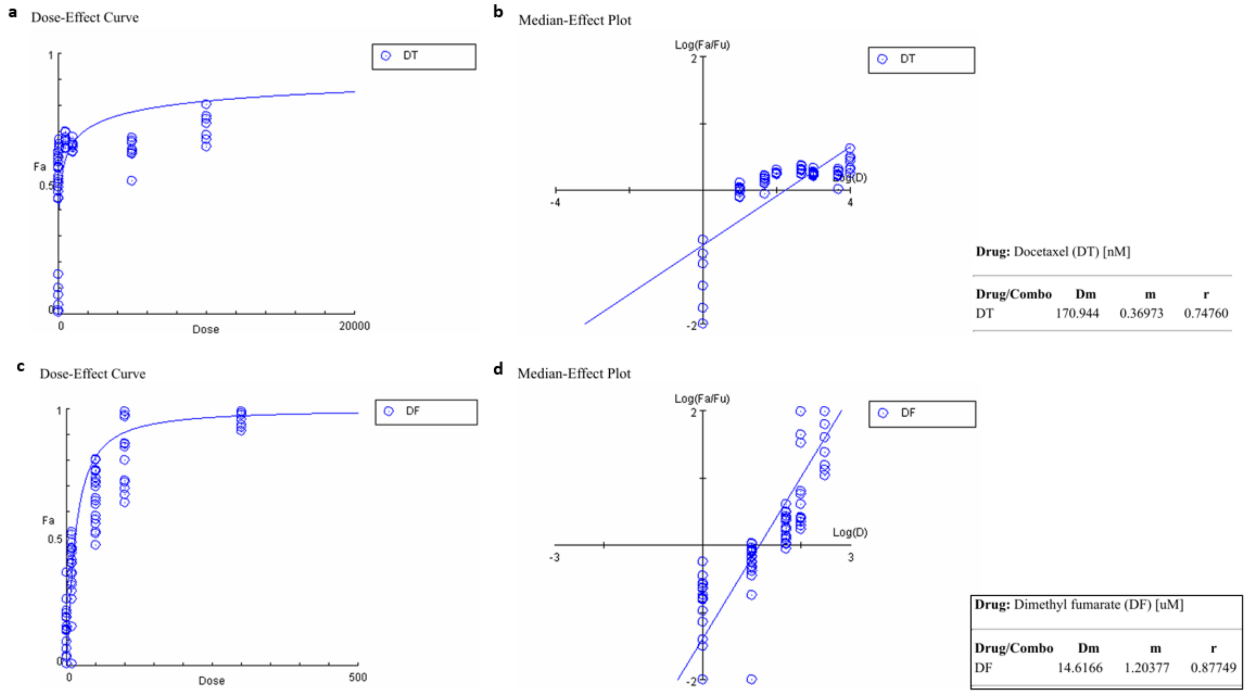

Supplementary Figure S3. Docetaxel and dimethyl fumarate median lethal concentration (LC_{50}) determination in PC3 cells. **(a)** Dose-effect curve plot for docetaxel, **(b)** median-effect plot for docetaxel, **(c)** Dose-effect curve for dimethyl fumarate, and **(d)** median-effect plot for dimethyl fumarate. DT: docetaxel; DF: dimethyl fumarate; Dm: median-effect dose equivalent to LC_{50}; m: slope; r: correlation coefficient. All data were determined with CompuSyn (version 1.0), n = 3.

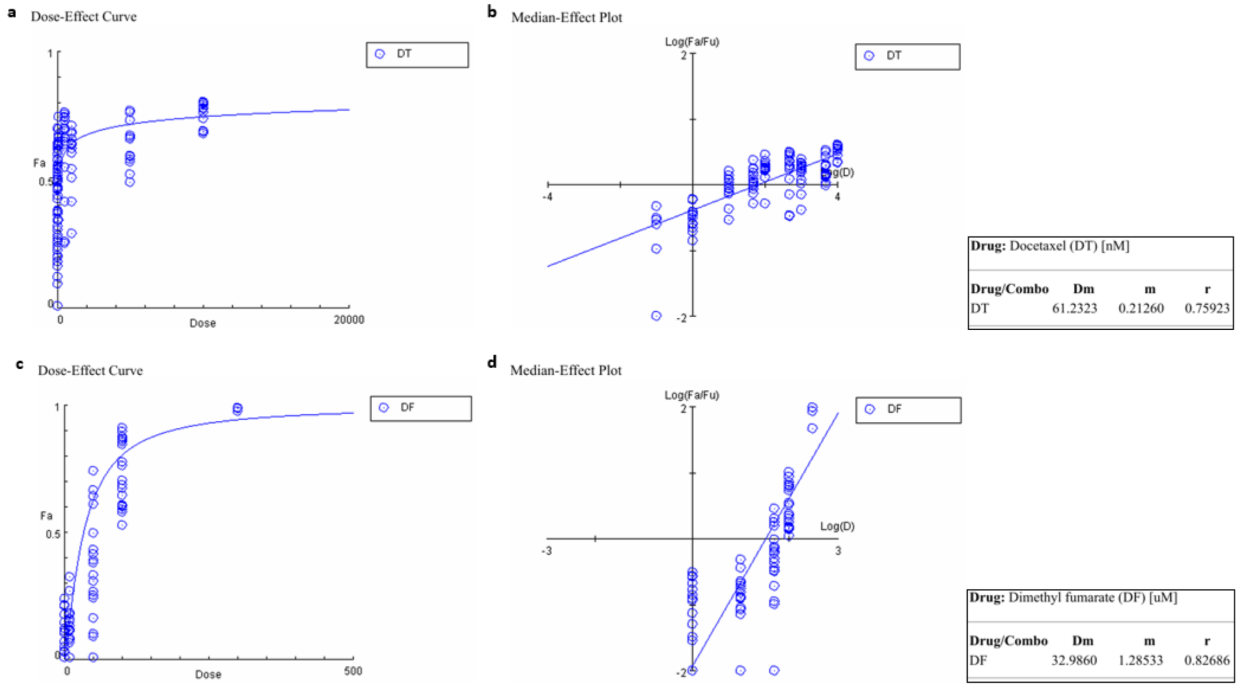

Supplementary Figure S4. Docetaxel and dimethyl fumarate median lethal concentration (LC_{50}) determination in LNCaP cells. **(a)** Dose-effect curve plot for docetaxel, **(b)** median-effect plot for docetaxel, **(c)** Dose-effect curve for dimethyl fumarate, and **(d)** median-effect plot for dimethyl fumarate. DT: docetaxel; DF: dimethyl fumarate; Dm: median-effect dose equivalent to LC_{50}; m: slope; r: correlation coefficient. All data were determined with CompuSyn (version 1.0), n = 3.

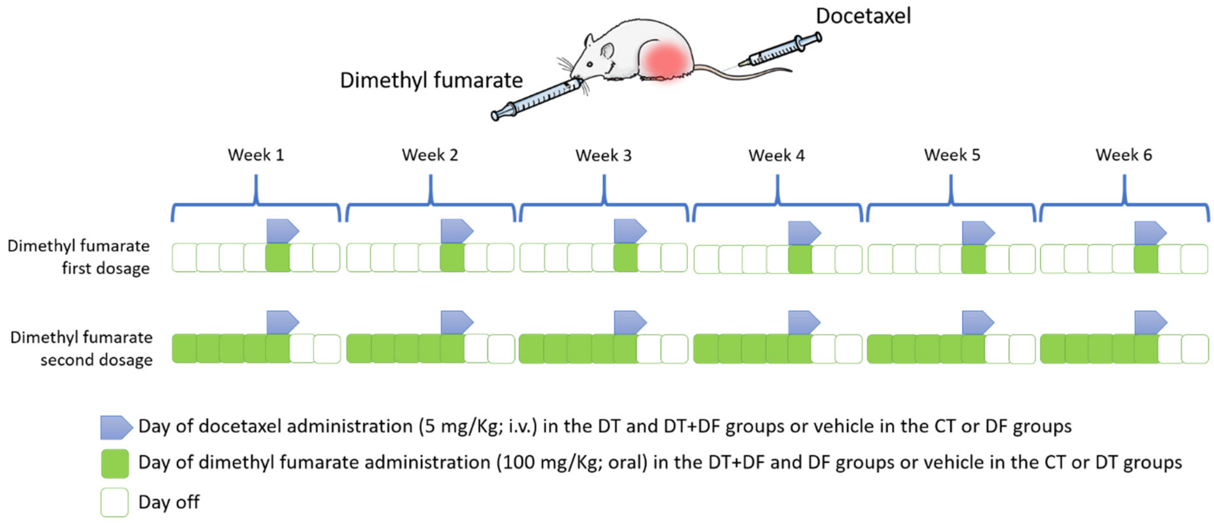

Supplementary Figure S5. Dimethyl fumarate regimens used with the model for the simultaneous induction of peripheral neuropathy and myelotoxicity by docetaxel in Wistar rats. The first dosage regimen consisted of a weekly oral dose of dimethyl fumarate (100 mg/kg) administered on the same day as intravenous docetaxel (5 mg/kg) for six weeks. The second dosage consisted of a dimethyl fumarate pre-treatment for four days before the dimethyl fumarate and docetaxel co-administration. The vehicles referred to are isotonic saline solution for docetaxel and carboxymethyl cellulose for dimethyl fumarate. CT: control group; DT: docetaxel group; DT+DF: docetaxel + dimethyl fumarate group; DF: dimethyl fumarate group; i.v.: intravenously.
